# Additive effects of mitral regurgitation on left ventricular strain in essential hypertensive patients as evaluated by cardiac magnetic resonance feature tracking

**DOI:** 10.3389/fcvm.2022.995366

**Published:** 2022-11-10

**Authors:** Si-Shi Tang, Rui Shi, Yi Zhang, Yuan Li, Xue-Ming Li, Wei-Feng Yan, Li Jiang, Zhi-Gang Yang

**Affiliations:** ^1^Department of Radiology, West China Hospital, Sichuan University, Chengdu, China; ^2^Department of Radiology, Chengdu Fifth People’s Hospital, Chengdu, China; ^3^Department of Radiology, Sichuan Cancer Hospital and Institute, Sichuan Cancer Center, School of Medicine, University of Electronic Science and Technology of China, Chengdu, China

**Keywords:** magnetic resonance imaging, hypertension, mitral valve insufficiency, left ventricular function, peak strain (PS)

## Abstract

**Objectives:**

Hypertension is one of the leading risk factors for cardiovascular disease. Mitral regurgitation (MR) is a heart valve disease commonly seen in hypertensive cases. This study aims to assess the effect of MR on left ventricle (LV) strain impairment among essential hypertensive cases and determine factors that independently impact the global peak strain of the LV.

**Materials and methods:**

We enrolled 184 essential hypertensive patients, of which 53 were patients with MR [HTN (MR +) group] and 131 were without MR [HTN (MR−) group]. Another group of 61 age-and gender-matched controls was also included in the study. All participants had received cardiac magnetic resonance examination. The HTN (MR +) group was classified into three subsets based on regurgitation fraction, comprising mild MR (*n* = 22), moderate MR (*n* = 19), and severe MR (*n* = 12). We compared the LV function and strain parameters across different groups. Moreover, we performed multivariate linear regression to determine the independent factors affecting LV global radial peak strain (GRS), circumferential peak strain (GCS), and global longitudinal peak strain (GLS).

**Results:**

HTN (MR−) cases exhibited markedly impaired GLS and peak diastolic strain rate (PDSR) but preserved LV ejection fraction (LVEF) compared to the controls. However, HTN (MR +) patients showed a decrease in LVEF and further deteriorated GRS, GCS, GLS, PDSR, and the peak systolic strain rate (PSSR) compared to the HTN (MR−) group and controls. With increasing degrees of regurgitation, the LV strain parameters were gradually reduced in HTN (MR +) patients. Even the mild MR group showed impaired GCS, GLS, PDSR, and PSSR compared to the HTN (MR−) group. Multiple regression analyses indicated that the degree of regurgitation was independently associated with GRS (β = -0.348), GCS (β = -0.339), and GLS (β = -0.344) in HTN (MR +) patients.

**Conclusion:**

GLS was significantly impaired in HTN (MR−) patients. MR may further exacerbate the deterioration of LV strain among essential hypertensive cases. Besides, the degree of regurgitation was independently correlated with GRS, GCS, and GLS in HTN (MR +) patients.

## Introduction

Hypertension is one of the leading risk factors for cardiovascular disease (1). Hypertensive heart disease is characterized by complex and diverse alterations in cardiac structure and function caused by hypertension (2, 3). Mitral regurgitation (MR) is a common heart valve disease in hypertensive cases (4). A longitudinal cohort study demonstrated that a 20 mmHg elevation in systolic blood pressure (SBP) was linked to a 26% higher risk of MR, while a 10-mmHg elevation in the diastolic blood pressure (DBP) was associated with a 24% higher risk of MR (5). MR may enhance left ventricular (LV) preload and aggravate myocardial damage of the hypertensive heart. Therefore, early evaluation of cardiac dysfunction in hypertensive patients with MR is crucial, which may facilitate the timely application of interventional measures, thus preventing adverse cardiovascular events and improving the prognosis.

Myocardial strain is an earlier and more sensitive indicator for subclinical myocardial dysfunction than LV ejection fraction (LVEF) (6, 7). It can provide early information on the diagnosis and therapy of myocardial damage (8–10). Moreover, the myocardial strain has been shown to have prognostic value for adverse cardiac outcomes (11–13). Cardiac magnetic resonance examination is regarded as the gold standard for non-invasively evaluating cardiac structure and function due to its strengths, such as high spatial resolution, multi-parametric, and multiplanar imaging (14). Recently, the routinely acquired cine sequence-based cardiac magnetic resonance feature-tracking has been applied to identify myocardial strain impairment (10, 15–17). However, to the best of our knowledge, cardiac magnetic resonance feature tracking is rarely utilized to detect the cumulative impact of MR on myocardial strain among hypertensive cases (9, 12). Therefore, this work compared LV strain and function among essential hypertensive cases with/without MR by cardiac magnetic resonance to investigate the effect of MR on LV myocardial strain impairment among hypertensive patients and identified risk factors that independently affected the LV global peak strain (PS).

## Materials and methods

This work was approved by the biomedical research ethics committee of our hospital. Informed consent was waived due to the retrospective nature of this investigation.

### Study population

From July 2012 to October 2021, 476 patients who were diagnosed with essential hypertension and had undergone cardiac magnetic resonance examination at our institution were enrolled in our study. The exclusion criteria were: ischemic heart disease, rheumatic heart disease, congenital heart disease, primary myocardiopathy, other valvular heart diseases, documented surgical procedures for heart diseases, image with artifacts caused by arrhythmia and the inability of adequate breath hold, leading to poor image quality inadequate for analysis, and incomplete key clinical data. Finally, 184 essential hypertensive cases aged 57.67 ± 14.10 years, with a body mass index (BMI) of 24.38 ± 3.39 kg/m^2^ were eligible and included in the study. Depending on whether the hypertensive patients were combined with MR upon detection by cardiac magnetic resonance, they were divided into hypertensive patients without MR [HTN (MR−) group] (131/184, 71.20%) and hypertensive patients with MR [HTN (MR +) group] (53/184, 28.80%). The study further included 61 patients of matched age and sex (average age of 55.27 ± 9.76 years; BMI of 22.63 ± 2.47 kg/m^2^) in the control group. The exclusion criteria for the control subjects were as follows: heart disease (coronary heart disease, valvular disease, etc.); chronic disease (hypertension, diabetes, hyperlipidemia, etc.); known systemic diseases; medication history. All the control subjects had also received cardiac magnetic resonance examination.

### Cardiac magnetic resonance protocol

All patients were examined in a supine position with a whole-body 3.0 T Siemens MAGNETOM Skyra scanner or a MAGNETOM Trio Tim system (Siemens Medical Solutions, Erlangen, Germany). This study also used the breath-holding technique and the standard ECG-triggering device throughout the process. All image data were acquired at the end of expiration. Furthermore, we performed cine imaging with the balanced steady-state free-precession sequence to acquire images in 8–12 continuous slices from the mitral valve to the LV apex in the short-axis view, as well as two, three, and four-chamber pictures in the long-axis view. The imaging parameters for the MAGNETOM Skyra scanner were: field of view-360 mm × 300 mm; matrix size-256 × 166; slice thickness-8 mm; temporal resolution-39.34 ms; repetition time-2.69 ms; echo time-1.2 ms; flip angle-38°. The imaging parameters for the MAGNETOM Trio Tim scanner were as follows: field of view-250 mm × 300 mm; matrix size-208 × 139; slice thickness-8 mm; temporal resolution-40.35 ms; repetition time-3.4 ms; echo time-1.31 ms; flip angle-50°.

### Cardiac magnetic resonance data analysis

#### Determination of cardiac volumetric and functional parameters

Two experienced cardiac radiologists with an experience of over 3 years analyzed the cardiac magnetic resonance images using commercially available offline software (cvi42, v. 5.11.2; Circle Cardiovascular Imaging Inc., Calgary, Alberta, Canada). They did not know the clinical information beforehand. This study manually outlined LV and the right ventricular (RV) endocardial/epicardial borders at the end-diastolic/end-systolic phases in each slice. The trabeculae and papillary muscles were eliminated. Then, the volumetric and functional parameters, including the LV/RV stroke volume (LVSV/RVSV), LV end-diastolic/end-systolic volume (LVEDV/LVESV), LV mass, and LVEF, were calculated automatically. Furthermore, we also indexed LVESV, LVEDV, LV mass, and LVSV for the body surface area (BSA) as LVESVI, LVEDVI, LVMI, and LVSVI, respectively, using the Mosteller formula (18).

#### Analysis of left ventricle strain

**FIGURE 1:**
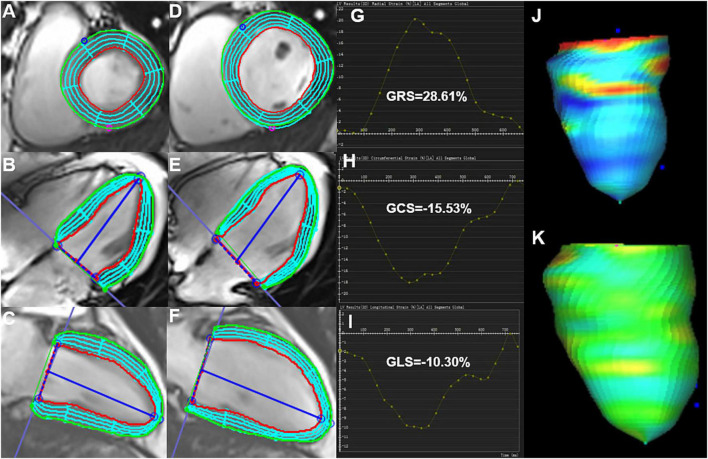
Cardiac magnetic resonance feature-tracking technology on cvi42 for the analysis of the LV strain. Manual drawing of the endocardium and epicardium of the LV at the end-systolic **(A–C)** and end-diastolic **(D–F)** phases. Then, the software automatically traced each voxel of the myocardium and calculated the LV strain parameters. **(G–I)** Measurement of the LV global peak strain parameters: GRS of 28.61%, GCS of −15.53%, and GLS of −10.31% were obtained. **(J,K)** 3D pseudo-color images for the LV end-systolic/end-diastolic GLS. LV, left ventricle; GRS, global radial peak strain; GLS, global longitudinal peak strain; GCS, global circumferential peak strain.

The LV short-axis, long-axis of horizontal four-chamber and vertical two-chamber cine images were loaded in the feature-tracking module of the cvi42 software. In addition, the epicardial/endocardial borders in all the above series were outlined at end-diastole and end-systole. Then, the LV global radial peak strain (GRS), global longitudinal peak strain (GLS), and global circumferential peak strain (GCS) (Figure 1), peak systolic strain rate (PSSR) in radial (PSSR-R), circumferential (PSSR-C), and longitudinal (PSSR-L) directions, and the peak diastolic strain rate (PDSR) in radial (PDSR-R), longitudinal (PDSR-L), circumferential (PDSR-C) directions were automatically acquired.

#### Evaluation of the mitral regurgitation fraction and mitral valve apparatus

MR was manifested as limited mitral valvular closure. An abnormal reversal of black blood flow was observed from LV to the left atrium *via* the mitral valve in the systolic stage in short-axis, two and three-chamber long-axis views. The regurgitation fraction (RF) was obtained using the formula RF = (LVSV-RVSV)/LVSV. Then, HTN (MR +) patients were classified into three subsets, mild (RF < 30%), moderate (30 ≤ RF < 50%), and severe regurgitation (RF ≥ 50%)(19, 20).

**FIGURE 2:**
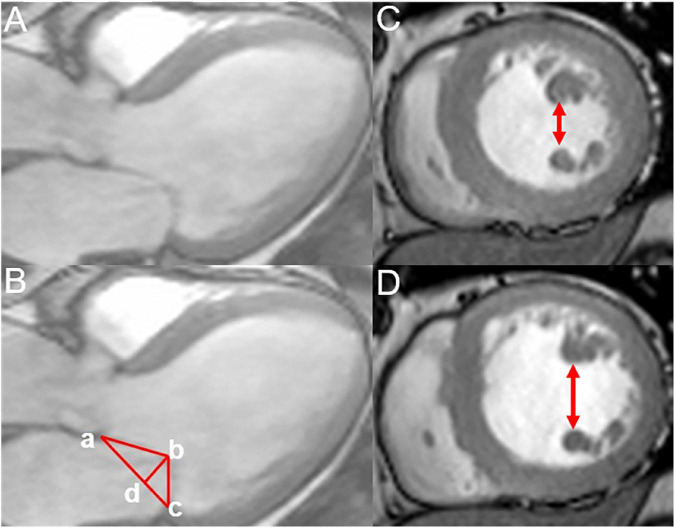
Measurement of the mitral annular geometric parameters and interpapillary muscle distance. **(A,B)** Measurement of the annular geometric parameters using cardiac magnetic resonance under a three-chamber view (mid-systolic); tethering height (b–d),mitral annular diameter (a–c), tethering area (enclosed by a–b–c), posterior tethering angle = ∠bca, and anterior tethering angle =∠bac. **(C,D)** Measurement of the interpapillary muscle distance during end-systolic and end-diastolic periods under the short axis.

We measured the geometric parameters for the mitral valve apparatus, including the mitral annular diameter (the linear distance between two ends of the mitral annulus), tethering height (the vertical distance between the coaptation of leaflets and the mitral annular plane), tethering area (the region surrounded *via* the annular plane and mitral leaflets), and the anterior and posterior tethering angles under the three-chamber view (mid-systolic). Besides, interpapillary muscle distance (IPMD), which was the distance between papillary muscle tips during the end-diastolic and end-systolic stages, was evaluated under the short-axis view (Figure 2) (21).

### Intra- and inter-observer reproducibility

The parameters of the LV strain and mitral valve apparatus of 80 randomized patients and the MR fraction of 30 randomized HTN (MR +) patients were evaluated by two independent observers blinded to clinical data to determine interobserver variability. One month later, one observer reanalyzed the parameters of the same subjects to determine the intra-observer variability.

### Statistical analysis

The statistical software SPSS version 24.0 (IBM Corp., Armonk, NY, USA) was used for statistical analysis. Categorical data, including sex, diabetic history, and types of antihypertensive drugs in the hypertensive and control groups, were presented as percentages and frequencies, while the chi-square test was applied for comparison among groups. Continuous variables, including age, BMI, SBP, DBP, resting heart rate, serum indices, LV function, strain, and mitral apparatus parameters, were presented as mean ± standard deviations if normally distributed and as median (25–75%, interquartile range) if not normally distributed. We compared the continuous variables among different groups using the one-way ANOVA along with the Bonferroni *post hoc* correction or the Kruskal–Wallis tests. Serum indexes were compared between two hypertensive groups using the student’s *t*-test. Spearman’s or Pearson’s correlation coefficient was calculated to examine the association of LV global PS with clinical indices and regurgitation degree. In the univariate analysis, variables with a *p*-value of < 0.1 and no collinearity in the sex, age, BMI, SBP, and history of diabetes were included in a multivariable backward linear regression analysis to evaluate the factors that independently predicted the LV strain among HTN (MR +) cases. Moreover, we determined the intraclass correlation coefficient (ICC) to evaluate the intra- and inter-observer variabilities. For all analyses, a *p*-value of < 0.05 (two-sided) indicated statistically significant differences between the groups.

## Results

### Baseline characteristics of the participants

No differences in sex, age, or resting heart rate were observed between the controls and hypertensive patients. There were also no differences in the history of diabetes, serum markers, and the use of antihypertensive drugs between HTN (MR−) and HTN (MR +) patients. However, HTN (MR−) and HTN (MR +) patients had significantly higher BMI than the control patients. The SBP and DBP in the HTN (MR−) group (137.0 ± 19.4 mmHg, 83.9 ± 13.9 mmHg) and HTN (MR +) group (137.3 ± 19.2 mmHg, 86.1 ± 14.7 mmHg) were significantly higher than the control group (116.0 ± 11.3 mmHg, 73.6 ± 7.8 mmHg) (all adjusted *P* < 0.05). Table 1 presents the baseline characteristics of all participants.

**TABLE 1:** Baseline features of the study cohort.

	Controls	HTN	
		
	(*n* = 61)	MR− (*n* = 131)	MR + (*n* = 53)
Sex, male (%)	36 (59.02%)	77 (58.78%)	28 (52.83%)
Age, years	55.27 ± 9.76	57.32 ± 14.27	58.52 ± 13.89
BMI, kg/m^2^	22.63 ± 2.47	24.39 ± 3.42*	24.34 ± 3.37*
SBP, mmHg	116.0 ± 11.3	137.0 ± 19.4*	137.3 ± 19.2*
DBP, mmHg	73.6 ± 7.8	83.9 ± 13.9*	86.1 ± 14.7*
Resting heart rate, bpm	71.0 ± 12.4	74.7 ± 14.4	69.5 ± 13.9
Diabetic history	-	26 (19.85%)	12 (22.64%)
TG, mmol/L	-	1.33 (0.97, 1.86)	1.37 (0.96, 2.29)
TC, mmol/L	-	4.30 ± 0.98	4.24 ± 1.19
HDL, mmol/L	-	1.30 ± 0.39	1.27 ± 0.35
LDL, mmol/L	-	2.50 ± 0.82	2.37 ± 0.92
Uric acid	-	335.00 (292.50, 407.50)	341.00 (265.13, 576.60)
eGFR, mL/min/1.73 m^2^	-	96.86 (83.06, 106.96)	84.34 (71.82, 107.93)
HTN with mild regurgitation, *n* (%)	-	-	22 (41.51%)
HTN with moderate regurgitation, *n* (%)	-	-	19 (35.85%)
HTN with severe regurgitation, *n* (%)	-	-	12 (22.64%)
ACEI/ARB, *n* (%)	-	55 (41.99%)	25 (47.17%)
Beta-blocker, *n* (%)	-	43 (32.82%)	11 (20.76%)
Calcium channel blocker, *n* (%)	-	74 (56.49%)	27 (50.94%)
Diuretics, *n* (%)	-	16 (12.21%)	12 (22.64%)

Data are presented as the number of patients (percentage) or as mean ± standard deviations.

HTN, hypertension; MR, mitral regurgitation; BMI, body mass index; SBP, systolic blood pressure; DBP, diastolic blood pressure; TG, triglycerides; TC, total cholesterol; HDL, high-density lipoprotein; LDL, low-density lipoprotein; eGFR, estimated glomerular filtration rate; ACEI, angiotensin-converting enzyme inhibitor; ARB, angiotensin II receptor blocker.

*Adjusted *P* < 0.05, HTN vs. control group.

### Comparison of left ventricle function and strain among hypertensive and control groups

The HTN (MR−) patients demonstrated higher LVMI, lower GLS and PDSR (-R, -C, -L) compared to controls (adjusted *P* < 0.05). Compared to the control and HTN (MR−) groups, the HTN (MR +) group showed higher LVEDVI, LVESVI, LVMI, and lower LVEF (adjusted *P* < 0.05). Furthermore, the HTN (MR +) group showed decreased PS, PDSR, and PSSR in every direction compared to the HTN (MR−) group and controls (adjusted *P* < 0.05). The detailed LV function and strain parameters are presented in Table 2.

**TABLE 2:** Comparison of cardiac magnetic resonance findings among hypertensive and control groups.

	Controls	HTN	
		
	(*n* = 61)	MR− (*n* = 131)	MR + (*n* = 53)
**LV function parameters**			
LVEDVI, mL/m^2^	71.61 (61.52, 77.43)	76.62 (61.53, 77.43)	114.66 (86.71, 145.81)*†
LVESVI, mL/m^2^	24.47 (19.32, 29.48)	27.48 (20.00, 30.07)	57.91 (30.80, 96.51)*†
LVSVI, mL/m^2^	46.38 (37.87, 52.10)	48.55 (39.89, 55.36)	53.84 (46.06, 60.77)
LVEF, %	63.52 ± 6.36	63.56 ± 7.20	48.88 ± 16.41*†
LVMI, g/m^2^	38.80 ± 13.60	54.14 ± 14.78*	70.67 ± 22.05*†
**LV strain parameters**			
**PS, %**			
GRS	33.93 (31.29, 40.87)	30.76 (26.08, 36.25)	17.72 (11.37, 30.65) *†
GCS	−20.56 ± 2.60	−19.89 ± 3.60	−14.66 ± 5.50*†
GLS	−14.72 ± 2.42	−12.38 ± 2.74*	−8.96 ± 3.58*†
**PSSR, 1/s**			
Radial	2.07 (1.63, 2.36)	1.82 (1.43, 2.27)	0.99 (0.66, 1.57)*†
Circumferential	−1.03 (−0.88, −1.20)	−1.00 (−0.88, −1.16)	−0.73 (−0.57, −0.94)*†
Longitudinal	−0.80 (−0.72, −0.93)	−0.75 (−0.60, −0.90)	−0.51 (−0.39, −0.71) *†
**PDSR, 1/s**			
Radial	−2.36 (−2.00, −2.92)	−1.91 (−1.44, −2.55)*	−1.04 (−0.63, −1.70)*†
Circumferential	1.25 (1.04, 1.41)	1.06 (0.86, 1.32)*	0.74 (0.56, 0.99)*†
Longitudinal	0.93 ± 0.33	0.76 ± 0.29*	0.55 ± 0.23*†

Data are presented as mean ± standard deviations or median (25%–75%, interquartile range).

HTN, hypertension; MR, mitral regurgitation; LV, left ventricular; EDV, end diastolic volume; ESV, end systolic volume; SV, stroke volume; EF, ejection fraction; M, mass; I, indexed to BSA; GRS, global radial peak strain; PS, peak strain; GCS, global circumferential peak strain; GLS, global longitudinal peak strain; PSSR, peak systolic strain rate; PDSR, peak diastolic strain rate.

*Adjusted *P* < 0.05, HTN vs. Control.

^†^Adjusted *P* < 0.05, HTN (MR +) vs. HTN (MR−).

### Comparison of left ventricle strain in hypertensive cases with different degrees of regurgitation

**FIGURE 3:**
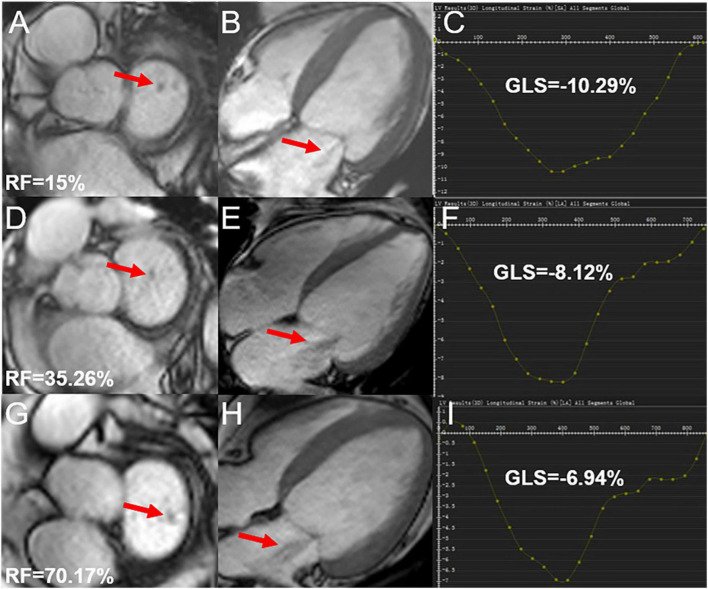
Cardiac magnetic resonance cine and GLS images in hypertensive cases showing mild, moderate, and severe degrees of regurgitation; **(A,B)** mild mitral regurgitation patient, male, 72-year-old, RF = 15%, LV short-axis **(A)**, 4-chamber **(B)** cine images demonstrating the regurgitation signal from the LV to LA (red arrow); **(D,E)** moderate mitral regurgitation patient, male, 52-year-old, RF = 35.26%, LV short-axis **(D)**, 4-chamber **(E)** cine images demonstrating the regurgitation signal from LV to LA (red arrow); **(G,H)** severe mitral regurgitation patient, male, 42 years old, RF = 70.17%, LV short-axis **(G)**, 4-chamber **(H)** cine images demonstrating abnormally reversed blood flow from LV to LA (red arrow); **(C,F,I)** the curves for LV GLS. RF, regurgitation fraction; GLS, global longitudinal peak strain; LV, left ventricle; LA, left atrium.

Among 53 HTN (MR +) patients, 22 (41.51%) showed mild regurgitation, 19 (35.85%) showed moderate regurgitation, and 12 (22.64%) showed severe regurgitation (Figure 3).

The GCS, GLS, PSSR-L, and PDSR-L in mild MR patients were decreased compared to HTN (MR−) patients (adjusted *P* < 0.05). The GRS, GCS, GLS, PSSR, and PDSR in all three directions in the moderate MR patients were lower than the HTN (MR−) patients. The moderate MR group showed lower GRS, GCS, GLS, PSSR-C, and PDSR-C than the mild MR group. The GRS, GCS, GLS, PDSR, and PSSR in all three directions were markedly reduced among the severe MR cases compared to the HTN (MR−) patients. Compared to the mild MR group, the GRS, GCS, GLS, PSSR-R, PDSR-R, PSSR-C, and PDSR-C of severe MR patients were markedly decreased (adjusted *P* < 0.05). The GRS, GCS, GLS, PSSR, and PDSR did not significantly differ between severe and moderate MR groups. More details of LV strain parameters among hypertensive patients are presented in Table 3.

**TABLE 3:** Comparison of LV strain in hypertensive cases with different degrees of regurgitation.

	HTN patients			
	
	without MR (*n* = 131)	mild MR (*n* = 22)	moderate MR (*n* = 19)	severe MR (*n* = 12)
LVEF, %	63.56 ± 7.20	56.53 ± 15.80*	48.04 ± 14.57*†	35.08 ± 10.55*†
**PS, %**				
GRS	30.76 (26.08, 36.25)	26.39 (14.83, 38.27)	13.49 (11.17, 26.87)*†	11.96 (9.75, 14.20)*†
GCS	−19.89 ± 3.60	−17.27 ± 5.35*	−13.47 ± 4.61*†	−11.11 ± 4.15*†
GLS	−12.38 ± 2.74	−10.52 ± 3.84*	−8.13 ± 2.96*†	−7.06 ± 2.57*†
**PSSR, 1/s**				
Radial	1.82 (1.43, 2.27)	1.37 (0.99, 1.97)	0.95 (0.62, 1.33) *	0.62 (0.56, 1.56) *†
Circumferential	−1.00 (−0.88, −1.16)	−0.92 (−0.72, −1.09)	−0.67 (−0.53, −0.92) *†	−0.60 (−0.52, −0.94) *†
Longitudinal	−0.75 (−0.60, −0.90)	−0.64 (−0.39, −0.79)*	−0.47 (−0.34, −0.71)*	−0.42 (−0.32, −0.56)*
**PDSR, 1/s**				
Radial	−1.91 (−1.44, −2.55)	−1.45 (−0.81, −2.19)	−1.04 (−0.62, −1.90)*	−0.75 (−0.50, −0.96)*†
Circumferential	1.06 (0.86, 1.32)	0.96 (0.68, 1.12)	0.74 (0.44, 0.93)*†	0.57 (0.53, 1.04)*†
Longitudinal	0.76 ± 0.29	0.59 ± 0.23*	0.51 ± 0.25*	0.51 ± 0.20*

Data are presented as mean ± standard deviations or median (25%–75%, interquartile range).

LV, left ventricular; HTN, hypertension; MR, mitral regurgitation; EF, ejection fraction; PS, peak strain; GRS, global radial peak strain; GCS, global circumferential peak strain; GLS, global longitudinal peak strain; PSSR, peak systolic strain rate; PDSR peak diastolic strain rate.

*Adjusted *P* < 0.05, HTN (MR +) vs. HTN (MR−).

^†^Adjusted *P* < 0.05, HTN with severe/moderate MR vs. HTN with mild MR.

### Comparison of mitral valve apparatus among different groups

All cases in the HTN (MR +) group showed higher mitral annular geometry parameters and IPMD (end-systolic/end-diastolic) compared to the patients of the HTN (MR−) and the control group. Differences between HTN (MR−) and control group were not significant. Moderate MR patients showed increased end-systolic/end-diastolic IPMD compared to mild MR patients. Severe MR patients showed higher mitral annular geometry parameters and IPMD than mild patients (adjusted *P* < 0.05). Differences between severe and moderate MR groups were not significant. The detailed mitral annular geometry parameters of hypertensive patients and controls are presented in Table 4.

**TABLE 4:** Comparison of mitral annular geometry and interpapillary muscle distance among different groups.

	Controls	HTN patients			
		
	(*n* = 61)	without MR (*n* = 131)	mild MR (*n* = 22)	moderate MR (*n* = 19)	severe MR (*n* = 12)
**Mitral annular geometry**					
Annulus diameter, mm	27.19 ± 2.28	27.72 ± 2.54	29.17 ± 1.58*	30.18 ± 2.81*	31.80 ± 2.16*†
Coaptation height, mm	7.15 (6.45, 7.80)	7.53 (6.86, 8.0)	8.44 (7.72, 9.05)*	8.79 (8.47, 9.58)*	9.34 (9.05, 9.73)*†
Tenting area, mm^2^	99.34 (93.29, 110.92)	102.13 (93.42, 112.80)	123.18 (112.71, 133.61)*	133.19 (120.44,136.53)*	141.47 (138.22, 159.89)*†
Anterior tethering angle°	23.01 ± 3.27	23.87 ± 3.15	25.46 ± 2.48*	27.11 ± 3.95*	29.10 ± 1.71*†
Posterior tethering angle°	33.76 (31.06, 35.88)	35.00 (32.54, 37.18)	37.97 (33.51, 39.00)*	38.05 (35.50, 41.42)*	41.11 (39.17, 42.47)*†
**IPMD**					
End-systolic, mm	8.27 ± 3.21	8.61 ± 2.83	12.40 ± 5.44*	15.78 ± 5.44*†	19.68 ± 4.30*†
End-diastolic, mm	20.04 (16.86, 21.46)	20.00 (16.83, 21.98)	22.72 (18.91, 25.26)*	23.96 (22.63, 27.06)*†	27.48 (25.70, 30.46)*†

Data are presented as mean ± standard deviations or median (25%–75%, interquartile range).

HTN, hypertension; MR, mitral regurgitation; IPMD, Interpapillary muscle distance.

*Adjusted *P* < 0.05, HTN (MR +) vs. HTN (MR−) and HTN (MR +) vs. controls.

^†^Adjusted *P* < 0.05, HTN with severe/moderate MR vs. HTN with mild MR.

### Independent predictive factors of left ventricle global peak strain among hypertensive patients showing mitral regurgitation

**FIGURE 4:**
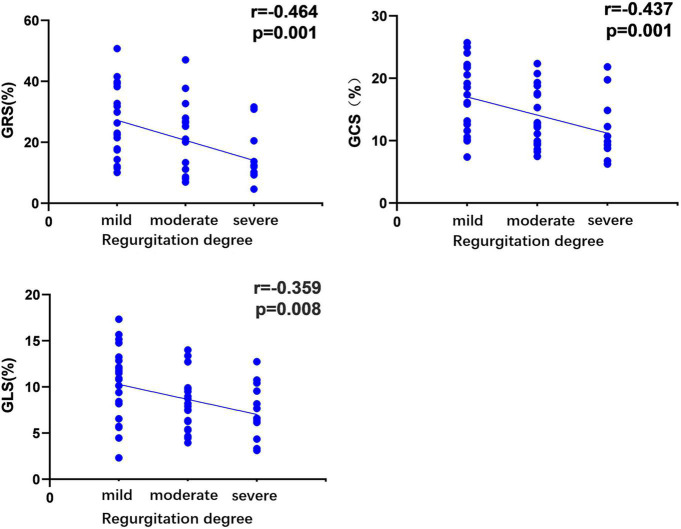
Correlation of regurgitation degree in hypertensive patients with left ventricular global peak strain. The absolute values of the circumferential and longitudinal-peak strain were analyzed to avoid the effect of the directional sign. r, correlation coefficient; GRS, global radial peak strain; GCS, global circumferential peak strain; GLS, global longitudinal peak strain.

Based on the univariate linear regression analysis, the regurgitation degree was negatively correlated with GRS (*R* = −0.464, *P* = 0.001), GLS (*R* = −0.359, *P* = 0.008), and GCS (*R* = −0.437, *P* = 0.001) (Figure 4). The smoking history was negative associated with GCS (*R* = −0.291, *P* = 0.043). Moreover, the level of triglycerides was negatively related to GCS (*R* = −0.357, *P* = 0.013) and GRS (*R* = −0.332, *P* = 0.023). According to the multivariate regression, the regurgitation degree independently predicted GRS (β = −0.348), GCS (β = −0.339), and GLS (β = −0.344) after adjusting the gender, age, BMI, SBP, and history of diabetes (Table 5).

**TABLE 5:** Univariate and multivariate analysis between clinical indexes and LV global PS among hypertensive patients showing MR.

	GRS			GCS			GLS		
			
	Univariate	Multivariate		Univariate	Multivariate		Univariate	Multivariate	
						
	*R*	β (R2 = 0.338)	*P*	*R*	β (R2 = 0.426)	*P*	*R*	β (R2 = 0.475)	*P*
MR degree	−0.464a	–0.348	0.029	−0.437a	–0.339	0.031	−0.359a	–0.344	0.018
SBP	0.136	0.068	0.671	0.107	0.117	0.479	–0.044	0.071	0.621
DBP	–0.076	−	−	–0.144	−	−	–0.236	−	−
Diabetes	0.095	0.272	0.108	–0.040	0.215	0.198	0.135	0.234	0.122
TG	−0.332*a*	–0.184	0.315	−0.357a	–0.071	0.726	−0.244b	–0.010	0.950
HR	–0.220	−	−	−0.245b	–0.123	0.506	–0.228	−	−
CH	–0.134	−	−	–0.144	−	−	–0.076	−	−
eGFR	0.274	−	−	0.233	−	−	0.044	−	−
Smoking	–0.175	−	−	−0.291a	–0.166	0.307	−0.271b	–0.015	0.923

a = *P* < 0.05; b = *P* < 0.1, factors with *P* < 0.1 and SBP, diabetic history were included in the multivariable backward linear regression after adjusting age, gender, body mass index.

LV, left ventricular; PS, peak strain; MR, mitral regurgitation; GRS, global radial peak strain; GCS, global circumferential peak strain; GLS, global longitudinal peak strain; SBP, systolic blood pressure; DBP, diastolic blood pressure; TG, triglycerides; HR, resting heart rate; CH, Coaptation height; eGFR, estimated glomerular filtration rate; R, correlation coefficient; β, regression coefficient; R2, coefficient of determination.

### Inter-and intra-observer variabilities

Excellent agreement between and within the observer was observed in the measurement of LV PS (ICC = 0.920–0.947 and 0.913–0.975, respectively), PSSR (ICC = 0.837–0.876 and 0.922–0.962, respectively), PDSR (ICC = 0.825–0.974 and 0.902–0.977, respectively), mitral annular geometry (ICC = 0.850–0.884 and 0.851–0.890, respectively), IPMD (ICC = 0.894–0.967 and 0.928–0.969, respectively) and MR fraction (ICC = 0.859 and 0.887, respectively) (Table 6).

**TABLE 6:** Intra-and inter-observer variabilities of LV strain, mitral annular geometry and mitral regurgitation fraction.

	Intra-observer		inter-observer	
		
	ICC	95% CI	ICC	95% CI
**PS**				
GRS	0.931	0.895–0.955	0.925	0.886–0.925
GCS	0.975	0.961–0.984	0.947	0.919–0.966
GLS	0.913	0.867–0.943	0.920	0.873–0.951
**PSSR**				
Radial	0.932	0.897–0.956	0.837	0.756–0.892
Circumferential	0.962	0.942–0.976	0.944	0.913–0.963
Longitudinal	0.922	0.882–0.950	0.876	0.814–0.919
**PDSR**	0.902	0.851–0.936	0.825	0.739–0.884
Radial	0.977	0.964–0.985	0.974	0.960–0.983
Circumferential	0.952	0.923–0.970	0.904	0.854–0.937
Longitudinal	0.932	0.897–0.956	0.837	0.756–0.892
**Mitral annular geometry**				
Annulus diameter	0.890	0.741–0.956	0.867	0.688–0.947
Coaptation height	0.873	0.698–0.949	0.850	0.651–0.940
Tenting area	0.853	0.658–0.941	0.875	0.704–0.950
Anterior tethering angle	0.867	0.615–0.946	0.872	0.552–0.956
Posterior tethering angle	0.851	0.579—-0.945	0.884	0.696–0.955
**Interpapillary muscle distance**				
End-systolic	0.969	0.915–0.989	0.967	0.907–0.988
End-diastolic	0.928	0.812–0.972	0.894	0.739–0.959
**Mitral regurgitation fraction**	0.887	0.778–0.945	0.859	0.724–0.930

LV, left ventricular; ICC, The intraclass correlation coefficients; CI, confidence interval; PS, peak strain; GRS, global radial peak strain; GCS, global circumferential peak strain; GLS, global longitudinal peak strain. PSSR, peak systolic strain rate; PDSR, peak diastolic strain rate.

## Discussion

In this study, we investigated the influence of MR on the function and strain in hypertension using cardiac magnetic resonance feature tracking. The main results were: (1) impaired GLS and PDSR, but preserved LVEF was observed in hypertensive patients without MR; (2) when hypertensive patients were concomitant with MR, significantly decreased LVEF and further deteriorated LV strain were observed in the radial, longitudinal and circumferential directions; (3) with deterioration of the degrees of regurgitation, LV strain was progressively reduced in hypertensive patients with MR, while the parameters of the mitral valve apparatus increased significantly; (4) the degree of regurgitation was independently correlated with GRS, GCS, and GLS.

Chronic mechanical stress from elevated blood pressure in hypertension can lead to LV remodeling, which involves LV cardiomyocyte hypertrophy and myocardial fibrosis, causing myocardial stiffness, decreased myocardial compliance, and consequent LV diastolic and systolic dysfunction (2, 3, 22, 23). In our study, the HTN (MR−) patients showed markedly reduced GLS compared to control patients, but the GCS was not significantly decreased, which was similar to the results of previous echocardiographic studies (24, 25). We hypothesized that LV myocardial fibrosis predominantly involved the subendocardial fiber. Therefore, the GLS, which mainly reflected the shortening of the subendocardial longitudinally oriented fiber, could deteriorate even in the early stages of hypertension. However, the GCS, which mainly represented the contractility of circumferential muscle fibers in the middle layer, remained spared, which could compensate for the longitudinal contractile dysfunction (10, 26). Therefore, traditional systolic indices, including LVEF, LVEDVI, LVESVI, and LVSVI, were preserved in HTN (MR−) patients in this study. These data were consistent with the findings of previous research (26, 27).

Cardiac magnetic resonance feature tracking is used to detect the diastolic function in the form of PDSR (16, 28). The PDSR in the HTN (MR−) patients was significantly impaired in all three directions compared to the control, whereas the PSSR, which represented the systolic data, was not significantly reduced. These data indicate that the abnormalities of diastolic function might occur before the systolic abnormalities in hypertension, which was consistent with previous research (29). Thus, our study adds to this body of evidence. In general, our research demonstrated that feature tracking technology might reveal subclinical LV dysfunction before traditional LVEF in hypertensive patients.

Hypertension may cause LV remodeling, resulting in LV geometric changes, papillary muscle shift, mitral annular dilation, change in the natural vertical angulation of the chordae tendineae, and tethering of the mitral leaflets, ultimately causing MR (30, 31). We observed that HTN (MR +) patients showed remarkably increased LVEDI, LVESI, LVMI, and decreased LVEF, PS, PDSR, and PSSR compared to controls and HTN (MR−) patients. The results indicated that MR might have a superimposed influence on LV function and strain in hypertensive patients, which is similar to the findings of a previous study (32). The pathological model may be that regurgitated volume in MR can increase LV preload, leading to LV remodeling, LV dilation, and eccentric hypertrophy, resulting in LV dysfunction (33). Significantly impaired LV dysfunction may occur under the double effect of hypertension and MR. Therefore, hypertensive patients with MR should be paid closer attention, and intervention should be made to prevent long-term adverse cardiac events.

When comparing the parameters of different degrees of MR regurgitation in hypertensive patients, it was observed that LVPS, PDSR, and PSSR were gradually reduced with the aggravation of the degree of MR regurgitation. Even the mild MR group showed impaired systolic and diastolic function compared to hypertensive patients without MR. Multivariate linear regression analysis indicated that the MR regurgitation degree was independently related to GRS, GCS, and GLS in HTN (MR +) patients. Our study demonstrated that the impairment of global PS possibly progressed with an increase in the degree of regurgitation. Several studies have reported that reduced GLS in patients with MR was correlated with LV dysfunction after intervention and higher risk for all-cause mortality (12, 34). Furthermore, the presence of MR may result in aggravated MR, which may be explained by the fact that MR−induced overload of the LV volume could induce LV dilatation, which puts more pressure on the mitral valve apparatus, causing further damage to the valve apparatus and aggravation of MR. In this situation, a vicious cycle between the ever-increasing LV volume and MR could be initiated (35). Therefore, even mild MR in hypertensive patients should be taken seriously.

A previous study reported that the diameter of the mitral annulus and IPMD were strongly correlated with the degree of MR regurgitation (21). This study observed that the parameters of mitral annular geometry and IPMD in groups with different degrees of MR were higher than those without MR. Compared to the mild MR group, the moderate MR group had higher end-systolic and end-diastolic IPMD, whereas the severe MR group showed an increase in mitral annular geometry and IPMD. Our results further suggest that the mitral valve apparatus might participate in the formation and aggravation of MR in hypertensive patients.

## Limitations

This study has certain limitations. First, this study was unicentric and cross-sectional, with a possible selection bias. Multi-center studies will be conducted in the future to validate the findings of this study further. Second, the lack of a follow-up in this study made the long-term impact of MR on the mortality of hypertensive patients unclear. This will be addressed in a future study. Third, no animal experiments were done in this study. Future research focusing on relevant pathological mechanisms will be carried out.

## Conclusion

Significant impairment of GLS was observed in hypertensive cases, and MR possibly deteriorated LV strain damage and cardiac insufficiency. The regurgitation degree was independently correlated with GCS, GRS, and GLS in the HTN (MR +) patients. Evaluation of LV strain in cardiac magnetic resonance feature tracking possibly assists clinicians in monitoring the development of cardiac deformation and facilitates additional therapies to delay LV myocardial damage among hypertensive cases developing MR.

## Data availability statement

The raw data supporting the conclusions of this article will be made available by the authors, without undue reservation.

## Ethics statement

The studies involving human participants were reviewed and approved by West China Hospital of Sichuan University Biomedical Research Ethics Committee. Written informed consent from the participants’ legal guardian/next of kin was not required to participate in this study in accordance with the national legislation and the institutional requirements. Written informed consent was not obtained from the minor(s)’ legal guardian/next of kin for the publication of any potentially identifiable images or data included in this article.

## Author contributions

S-ST, RS, LJ, and Z-GY designed the research and wrote and reviewed the manuscript. S-ST, RS, YZ, YL, Z-GY, and LJ performed the experiments. S-ST, RS, LJ, YZ, X-ML, and W-FY collected and sorted statistical data. W-FY, YL, X-ML, and Z-GY analyzed the data. All authors read and approved the final manuscript.

## Abbreviations

MR: mitral regurgitation
LV: left ventricular
HTN (MR +): hypertensive patients with mitral regurgitation
HTN (MR−): hypertensive patients without mitral regurgitation
PS: peak strain
GRS: global radial peak strain
GCS: global circumferential peak strain
GLS: global longitudinal peak strain
PSSR: peak systolic strain rate
PDSR: peak diastolic strain rate
LVEF: left ventricular ejection fraction
BMI: body mass index
SBP: systolic blood pressure
DBP: diastolic blood pressure
LVSV: left ventricular stroke volume
RVSV: right ventricular stroke volume
LVEDV: left ventricular end-diastolic volume
LVESV: left ventricular end-systolic volume
BSA: body surface area
LVEDVI: left ventricular end-diastolic volume indexed to body surface area
LVESVI: left ventricular end-systolic volume indexed to body surface area
LVSVI: left ventricular stroke volume indexed to body surface area
LVMI: left ventricular mass indexed to body surface area
RF: regurgitation fraction
IPMD: interpapillary muscle distance
ICC: intraclass correlation coefficient.
